# The importance of baseline fractional flow reserve to detect significant coronary artery stenosis in different patient populations

**DOI:** 10.5830/CVJA-2023-045

**Published:** 2023-09-21

**Authors:** Ismet Zengin, Alper Karakus

**Affiliations:** Department of Cardiology, Bursa City Hospital, Health Sciences University, Bursa, Turkey; Department of Cardiology, Bursa Yüksek Ihtisas Training and Research Hospital, Health Sciences University, Bursa, Turkey

**Keywords:** fractional flow reserve, coronary artery disease, baseline Pd/Pa, adenosine

## Abstract

**Introduction:**

Fractional flow reserve (FFR) assessment compares the blood flow on either side of a blockage in the coronary artery and indicates how severe the stenosis is in the artery. Intravenous adenosine is widely used to achieve conditions of stable hyperaemia for the measurement of FFR. However, intravenous adenosine affects both systemic and coronary vascular beds differentially. Therefore, FFR has some limitations, such as the side effects of adenosine and the long procedure time. In addition, there are not enough studies on the evaluation of the baseline ratio of distal coronary pressure to aortic pressure (Pd/Pa) according to standard cut-off values in coronary stenosis under special clinical conditions. This study aimed to assess the diagnostic power of the baseline FFR value for critical coronary stenosis and to determine its predictive value in special patient groups.

**Methods:**

This retrospective study included 158 patients, who were stratified as Q1 (< 0.89), Q2 (0.89–0.92), Q3 (0.93–0.95) and Q4 (> 0.95) based on baseline FFR values. The baseline Pd/Pa value, the change in adenosine FFR and the raw FFR change were recorded. Its predictive value was also calculated for specific patient groups.

**Results:**

The threshold value of baseline FFR level for predicting critical stenosis was ≤ 0.92 with a sensitivity of 92.8% and a specificity of 82% (upper limit of Q2 cartilage). Patients with a baseline FFR value ≤ 0.92 had a 58.4-fold greater likelihood of a critical outcome compared with patients with a baseline FFR value > 0.92 (OR: 58.4; 95% CI: 20.3–124.6). In patients with a baseline FFR ≤ 0.92, the Q1 group had a 10.23-fold higher odds of critical stenosis compared with the Q2 group (OR: 10.23; 95% CI: 2.14–48.84). The same values had similar diagnostic power for all specific patient groups.

**Conclusion:**

The baseline FFR value could be used to predict critical coronary stenosis in our patients and had similar value for predicting lesion severity in all the subgroups.

Clinical studies and evidence on fractionated flow reserve (FFR), which is widely used in the physiological assessment of coronary stenosis, are growing more numerous. Intravenous adenosine is widely used to achieve conditions of stable hyperaemia for the measurement of FFR. If we review the results of FFR studies, we see that the researchers have used different methods, which gave different results. This can lead to confusion in clinical practice. In addition, certain agents may create some risks to patients. For instance, the commonly used adenosine has side effects, including transient atrioventricular (AV) block, chest pain and dyspnoea.^1^ In adenosine FFR, depending on the route of administration (for example, intravenous infusion), the duration of the procedure may be prolonged and patients may be exposed to risks related to the longer duration.

Given these limitations of FFR, other methods of assessment have come to the forefront. One of these is measuring the baseline ratio of the coronary pressure distal to the coronary lesion to the aortic pressure (Pd/Pa), which is performed without the use of a hyperaemic agent. There are studies evaluating the correlation of the Pd/Pa ratio with the FFR value using hyperaemic agents.^1^-^3^ In parallel, the instantaneous wave-free ratio (iFR), which is a haemodynamic assessment that makes use of initial pressure values, requires separate equipment and software, which may not be cost effective. It is generally accepted that the Pd/Pa ratio, which can be expressed as the basal FFR value, is not influenced by many parameters. Nevertheless, there are not enough subgroup analyses of basal FFR measurements.

Data on the use of Pd/Pa values obtained from previous studies in different patient populations are lacking. The aim of this study was to evaluate the diagnostic power of the baseline FFR value for critical coronary stenosis and to determine its predictive value in special patient groups.

## Methods

This retrospective study included 158 patients with 158 lesions who underwent FFR measurement between July 2019 and January 2023 at the Bursa City Hospital, Bursa, Turkey. FFR was planned to be performed in patients with acute coronary syndrome five days after treatment of the responsible lesion or in patients with chronic coronary syndrome with persistent symptoms despite optimal medical treatment and with 50–90% anatomical stenosis on conventional coronary angiography (CAG).

Exclusion criteria were New York Heart Association (NYHA) class IV heart failure, cardiogenic shock, severe liver disease, respiratory failure necessitating intubation, severe obstructive airway disease, active malignancy, sepsis/septic shock and active infection, and history of allergy to iodinated contrast agents, adenosine or adenosine-containing products.

Demographic and clinical characteristics, medical history, cardiovascular risk factors, laboratory tests, electrocardiography, echocardiography, CAG, stent length and diameter if undergoing percutaneous coronary intervention (PCI) were collected.

Diabetes mellitus (DM) was defined as a diagnosis of fasting blood glucose ≥ 126 mg/dl (6.99 mmol/l) or glycated haemoglobin (HbA1c) ≥ 6.5% or the use of diabetes medication. Hypertension (HT) was defined as a diagnosis of systolic blood pressure ≥ 140 mm/Hg, diastolic blood pressure ≥ 90 mm/Hg or use of antihypertensive medication. Smoking was defined as current smoking or quitting within the previous year. Previous myocardial infarction (MI) was defined as MI diagnosed according to the European Society of Cardiology Fourth Universal Definition of Myocardial Infarction (2018) guidelines and stent implantation for an occlusive epicardial coronary lesion.^4^ An estimated glomerular filtration rate (eGFR) < 60 ml/min/1.73 m^2^ for three months or longer was defined as chronic kidney disease (CKD).^5^

The study adhered to the tenets of the Declaration of Helsinki and was conducted with the permission of the Clinical Ethical Research Committee of Bursa City Hospital.

FFR measurement was planned for patients with anatomical stenosis between 50 and 90% on CAG. Using the Boston Polaris multimodality guidance system, the COMET I or COMET II pressure guidewire was first flushed and reset with the pressure sensor externally placed in a horizontal position at the heart level. The guidewire pressure was then equalised with aortic pressure. The guidewire sensor was passed through the lesion at least 5 mm distal to the narrowed segment, and baseline Pd/Pa was measured and recorded as the baseline FFR value.

Intracoronary isosorbide dinitrate was administered at a dose of 200 μg to rule out vasoconstriction,^6^ and adenosine infusion was then begun at a dose of 140 μg/kg/min via a large peripheral venous access. The lowest Pd/Pa value at the time of maximal hyperaemia was measured between 30 and 120 seconds after the start of the infusion, and this value was recorded as the FFR value. The guidewire was pulled proximal to the lesion and into the aorta. An increase in pressure and Pd/Pa value was observed, and the success of the procedure was confirmed. Values < 0.75 were considered critical stenosis. Values of 0.75–0.80 were decided by individual assessment. For values > 0.80, deferring PCI was considered.

After collecting the data from the intracoronary pressure measurement protocols, the correlation of FFR values with various parameters (DM, HT, history of previous MI, vessel diameter, stent length, stent diameter) was assessed according to the presence or absence of critical coronary stenosis. Patients were divided into four groups, Q1, Q2, Q3 and Q4 (< 0.89, 0.89–0.92, 0.93–0.95 and > 0.95, respectively), taking into account patients’ baseline Pd/Pa values and clinical characteristics, including DM, HT, multivessel disease, previous MI, CKD and vessel diameter (< 3 mm or > 3 mm), stent length and stent diameter. Changes in FFR value at the time of maximal hyperaemia compared to baseline FFR value were evaluated, including subgroup analysis.

## Statistical analysis

Statistical analysis was performed using the Statistical Package for the Social Sciences (SPSS version 21.0; IBM Corp, Armonk, NY, USA). Statistical significance was set at p < 0.05. The Shapiro–Wilk test was used to assess whether the measurements conformed to a normal distribution. The Student’s t-test or Mann–Whitney U-test was used for continuous variables according to the distribution pattern. Categorical variables are presented as percentages and the chi-squared test was used for comparison. A receiver operating characteristic (ROC) curve was used for sensitivity and specificity analysis.

## Results

The mean patient age was 59.8 ± 9.2 years and 124 patients were male (78.5%). There was no significant difference in risk factors between the Q1, Q2, Q3 and Q4 groups. Multivessel disease was significantly higher in the Q1 group (p = 0.041). Baseline FFR value was lower in the Q1 group than in the other groups. Adenosine values were significantly lower in the Q1 and Q2 groups than in the Q3 and Q4 groups (p < 0.001). When analysing the percentage change, the Q3 and Q4 groups showed a greater change in adenosine level than the Q1 and Q2 groups (p < 0.001).

**Fig. 1:**
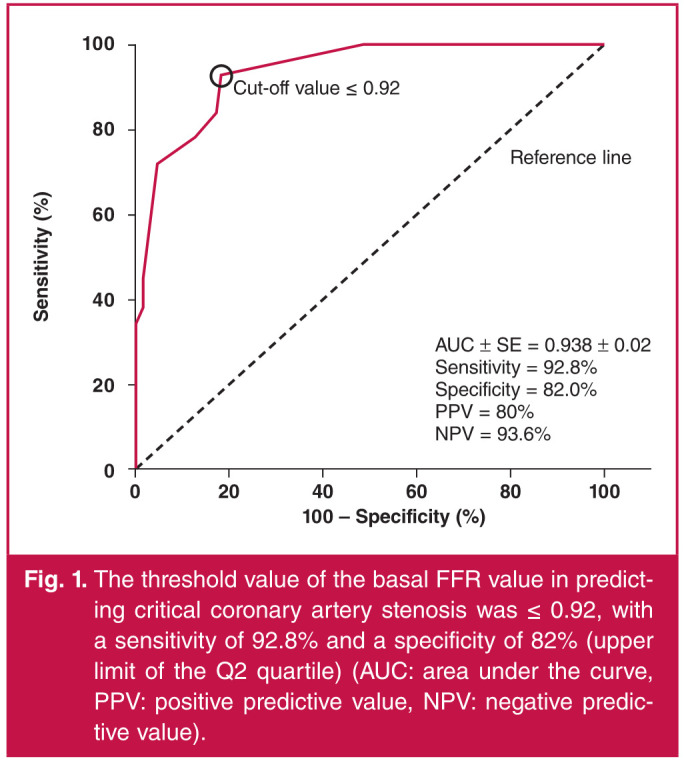
The threshold value of the basal FFR value in predicting critical coronary artery stenosis was ≤ 0.92, with a sensitivity of 92.8% and a specificity of 82% (upper limit of the Q2 quartile) (AUC: area under the curve, PPV: positive predictive value, NPV: negative predictive value).

**Fig. 2:**
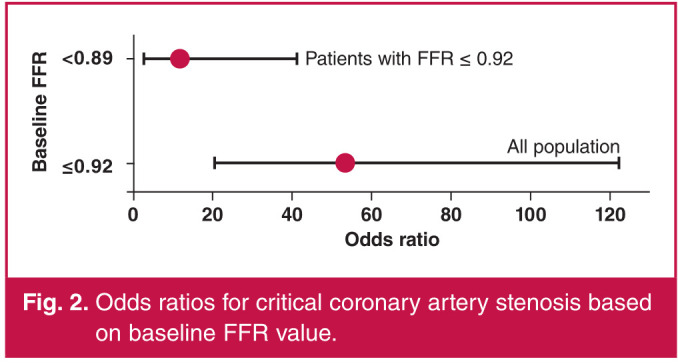
Odds ratios for critical coronary artery stenosis based on baseline FFR value.

Based on the FFR results, critical stenosis was more common in the Q1 and Q2 groups, whereas non-critical stenosis was more common in the Q3 and Q4 groups (p < 0.001). The threshold value of baseline FFR level for predicting critical stenosis was ≤ 0.92 with a sensitivity of 92.8% and a specificity of 82% (upper limit of Q2 quartile). Patients with a baseline FFR value ≤ 0.92 had a 58.4-fold greater likelihood of a critical outcome compared with patients with a baseline FFR value > 0.92 (OR: 58.4; 95% CI: 20.3–124.6). In patients with a baseline FFR ≤ 0.92, the Q1 group had a 10.23-fold higher odds of critical stenosis compared with the Q2 group (OR: 10.23; 95% CI: 2.14–48.84) (Figs 1, 2).

The number of patients with vessel diameter > 3 mm was significantly lower in the Q3 and Q4 groups (p < 0.001). No statistically significant difference was found between the four groups in other parameters (Table 1). The results related to FFR value after adenosine infusion showed that 89 patients had non-critical stenosis and 69 had critical stenosis. HT was more common in the critical stenosis group (p = 0.036).

**Table 1:** Demographic information and clinical findings of the study population

			*Baseline*	*FFR*		
*Variables*	*All population (n = 158)*	*Q1 0.89) (n = 40)*	*Q2 (0.89-0.92) (n = 40)*	*Q3 (0.93-0.95) (n=39)*	*Q4 -0.95) = 39)*	*p-value*
Age, years	59.8 + 9.2	60.1 + 10.2	59.4 + 9.9	61.6 + 8	58.3 + 8.4	0.478
Male gender, n (%)	124 (78.5)	33 (82.5)	34 (85.0)	(71.8)	(74.4)	0.433
Diabetes mellitus, n (%)	44 (27.8)	13 (32.5)	11 (27.5)	7 (17.9)	(33.3)	0.399
Hypertension, n (%)	(38.6)	20 (50.0)	(37.5)	(38.5)	11 (28.2)	0.271
Culprit lesion, n (%)						
LAD	115(72.8)	5 (87.5)	4(85.0)	(76.9)	16 (41.0)	
Cx	18 (11.4)	2 (5.0)	(2.5)	5 (12.8)	(25.6)	
RCA	23 (14.6)	3 (7.5)	5 (12.5)	4 (10.3)	(28.2)	< 0.001*
LMCA	1 (0.6)	0	0	0	1 (2.6)	
D1	(0.6)	0	0	0	1 (2.6)	
Segment, n (%)						
Ostial	45 (28.5)	14 (35.0)	11 (27.5)	11 (28.2)	9 (23.1)	
Proximal	64 (40.5)	16 (40.0)	17 (42.5)	11 (28.2)	20 (51.3)	
Mid	46 (29.1)	10 (25.0)	11 (27.5)	17 (43.6)	8 (20.5)	0.264
Distal	3 (1.9)	0	1 (2.5)	0	2 (5.1)	
Multivessel, n (%)	97 (61.4)	32 (80.0)	21 (52.5)	(56.4)	(56.4)	0.041*
History of MI, n (%)	64 (40.5)	16 (40.0)	(40.0)	18 (46.2)	(35.9)	0.827
CTO, n (%)	10 (6.3)	6 (15.0)	(2.5)	(2.6)	(5.1)	0.118
FFR. %						
Baseline	91.5 + 4.8	84.9 + 2.2	90.2 + 1.1	93.7 + 1.0	97.6 + 1.2	<0.001*
Adenosine	82.7 + 8.0	74.2 + 4.6	79.6 + 5.2	85.8 + 5.1	91.6 + 4.0	<0.001*
Change	9 (5.2-13)	11.6 (9.6-14.4)	12.7 (6.7-16)	7.4 (4.2-10.8)	5.2 (3.1-8.2)	<0.001*
Lesion status. n (%)						
Non-critical		(5.0)	(35.0)	34 (87.2)	(100.0)	
Critical		(95.0)	(65.0)	5 (12.8)	0	<0.001*
Vessel diameter. n (%)						
< 3 mm	70 (44.3)	11(27.5)	(27.5)	(59.0)	25 (64.1)	
> 3 mm	88 (55.7)	(72.5)	29 (72.5)	(41.0)	14(35.9)	< 0.001*
Stent size, mm	3 (2.8-3)	3 (2.9-3.0)	3 (2.8-3.0)	3 (2.8-3.0)		0.858
Stent length, mm	28 (20-40)	32 (28-40)	24 (16-33)	20 (16-52)		0.310
GFR, ml/min/1.73 m²	87.9 + 20.9	89.7 + 20.2	92.1 + 17.3	78.3 + 23.2	90.1 2 21.7	0.150
Creatinine, mg/dl	0.8 (0.7-1.0)	0.9 (0.8-1.0)	0.9 (0.7-1.0)	0.9 (0.8-1.1)	0.8 (0.7-0.9)	0.282
Haemoglobin, g/dl	13.7 + 2.1	13.6 + 2.3	14.0 + 2.0	12.9 + 1.8	14.5 + 2.1	0.142
LVEDD, mm	50.1 + 5.2	51.7 + 5.4	51.2 + 3.9	46.3 + 4.1	45.3 + 2.1	0.081
LVESD, mm	34.1 + 5.9	34.9 + 4.6	37.3 + 10.1	32.3 + 7.4	31.0 + 2.6	0.554
LVEF, %	52.9 + 9.3	51.8 + 9.3	52.1 + 8.7	55.3 + 7.5	53.1 + 11.8	0.633

Data are presented as mean ± SD or median (IQR) or number (%).

*p < 0.05 indicates statistical significance. Bold characters show differences between groups.

LAD: left anterior descending, Cx: circumflex, RCA: right coronary artery, LMCA: left main coronary artery, D1: diagonal 1, MI: myocardial infarction, CTO: chronic

total oclusion, FFR: fractionated flow reserve, GFR: glomerular filtration rate, LVEDD: left ventricular end-diastolic diameter, LVESD: left ventricular end-systolic

diameter, LVEF: left ventricular ejection fraction.

The number of FFRs performed for right coronary artery (RCA) lesions was higher in the group with non-critical stenosis (p < 0.001). Multivessel disease was also more common in the critical stenosis group (p = 0.012). In the critical stenosis group, there was a significant difference in baseline FFR value, adenosine FFR value and percentage change in FFR compared to the non-critical stenosis group (p < 0.001).

While < 3-mm vessel diameter was more common in the non-critical stenosis group, > 3-mm vessel diameter was more common in the critical stenosis group (p < 0.001). Left ventricular end-diastolic diameter (LVEDD) was also significantly higher in the critical stenosis group (p = 0.025). Otherwise, no significant differences were found between the two groups (Table 2).

**Table 2:** Findings associated with critical FFR outcome after adenosine

*Variables*	*Non-critical (n=89)*	*Critical (n=69)*	*p-value*
Age, years	60.3 + 8.7	59.3 + 9.7	0.512
Male gender, n (%)	68 (76.4)	56 (81.2)	0.560
Diabetes mellitus, n (%)	(27.0)	(29.0)	0.858
Hypertension, n (%)	(31.5)	33 (47.8)	0.036*
Culprit lesion, n (%)			
LAD	54 (60.7)	61 (88.4)	
Cx	16 (18.0)	2 (2.9)	
RCA	(19.1)	6 (8.7)	< 0.001*
LMCA	(1.1)	0	
D1	(1.1)	0	
Segment, n (%)			
Ostial	22 (24.7)	23 (33.3)	
Proximal	35 (39.3)	29 (42.0)	0.430
Mid	30 (33.7)	16 (23.2)	
Distal	2 (2.2)	1 (1.4)	
Multivessel, n (%)	(52.8)	50 (72.5)	0.012*
History of MI, n (%)	35 (39.3)	29 (42.0)	0.731
CTO, n (%)	3 (3.4)	7 (10.1)	0.160
FFR, %			
Baseline	94.7 + 3.0	87.5 + 3.6	<0.001*
Adenosine	88.8 + 4.3	74.8 + 4.0	<0.001*
Change	5.5 (3.3-8.2)	13 (10.7-16.7)	<0.001*
Vessel diameter, n (%)			
< 3 mm	59 (66.3)	1 (15.9)	<0.001*
> 3 mm	30 (33.7)	58 (84.1)	
Stent size, mm	2.9 (2.8-3)	3 (2.9-3)	0.554
Stent length, mm	18 (16-20)	28 (20-40)	0.171
GFR, ml/min/1.73 m²	83.3 + 22.6	91.5 + 18.8	0.068
Creatinine, mg/dl	0.9 (0.8-1.0)	0.8 (0.7-1.0)	0.335
Haemoglobin, g/dl	13.9 + 2.0	13.6 + 2.2	0.551
LVEDD, mm	46.8 + 3.9	51.5 + 5.1	0.025*
LVESD, mm	31.1 + 4.6	35.8 + 6.1	0.096
LVEF, %	54.5 + 9.8	51.6 + 8.7	0.154

Data are presented as mean ± SD or median (IQR) or number (%).

*p < 0.05 indicates statistical significance.

LAD: left anterior descending, Cx: circumflex, RCA: right coronary artery,

LMCA: left main coronary artery, D1: diagonal 1, MI: myocardial infarction,

CTO: chronic total oclusion, FFR: fractionated flow reserve, GFR: glomerular

filtration rate, LVEDD: left ventricular end-diastolic diameter, LVESD: left

ventricular end-systolic diameter, LVEF: left ventricular ejection fraction.

In the non-critical stenosis group, the change in FFR value after adenosine infusion was greater in patients with multivessel disease than in those with single-vessel disease (p = 0.008), and in those with chronic total occlusion (CTO) (p = 0.037). In the Q3 group, this change was higher in those with critical stenosis (p = 0.001). In the non-critical stenosis group, this change was higher in those with a vessel diameter > 3 mm (p = 0.027). No significant difference was found in the other parameters (Table 3).

**Table 3:** Findings associated with change in FFR after adenosine

*Variables*	*Non-critical (n=89)*	*Critical (n=69)*	*p-value*
Age, years	60.3 + 8.7	59.3 + 9.7	0.512
Male gender, n (%)	68 (76.4)	56 (81.2)	0.560
Diabetes mellitus, n (%)	(27.0)	(29.0)	0.858
Hypertension, n (%)	(31.5)	33 (47.8)	0.036*
Culprit lesion, n (%)			
LAD	54 (60.7)	61 (88.4)	
Cx	16 (18.0)	2 (2.9)	
RCA	(19.1)	6 (8.7)	< 0.001*
LMCA	(1.1)	0	
D1	(1.1)	0	
Segment, n (%)			
Ostial	22 (24.7)	23 (33.3)	
Proximal	35 (39.3)	29 (42.0)	0.430
Mid	30 (33.7)	16 (23.2)	
Distal	2 (2.2)	1 (1.4)	
Multivessel, n (%)	(52.8)	50 (72.5)	0.012*
History of MI, n (%)	35 (39.3)	29 (42.0)	0.731
CTO, n (%)	3 (3.4)	7 (10.1)	0.160
FFR, %			
Baseline	94.7 + 3.0	87.5 + 3.6	<0.001*
Adenosine	88.8 + 4.3	74.8 + 4.0	<0.001*
Change	5.5 (3.3-8.2)	13 (10.7-16.7)	<0.001*
Vessel diameter, n (%)			
< 3 mm	59 (66.3)	1 (15.9)	<0.001*
> 3 mm	30 (33.7)	58 (84.1)	
Stent size, mm	2.9 (2.8-3)	3 (2.9-3)	0.554
Stent length, mm	18 (16-20)	28 (20-40)	0.171
GFR, ml/min/1.73 m²	83.3 + 22.6	91.5 + 18.8	0.068
Creatinine, mg/dl	0.9 (0.8-1.0)	0.8 (0.7-1.0)	0.335
Haemoglobin, g/dl	13.9 + 2.0	13.6 + 2.2	0.551
LVEDD, mm	46.8 + 3.9	51.5 + 5.1	0.025*
LVESD, mm	31.1 + 4.6	35.8 + 6.1	0.096
LVEF, %	54.5 + 9.8	51.6 + 8.7	0.154

LAD: left anterior descending, Cx: circumflex, RCA: right coronary artery, MI: myocardial infarction, CTO: chronic total oclusion, FFR: fractionated flow reserve, GFR: glomerular filtration rate, LVEDD: left ventricular end-diastolic diameter, LVESD: left ventricular end-systolic diameter, LVEF: left ventricular ejection fraction.

## Discussion

The findings of this study suggest that (1) the baseline FFR value can be used to predict the presence of critical coronary stenosis and it can be accepted at a cut-off value of ≤ 0.92 with a sensitivity of 92.8% and a specificity of 82%; (2) according to the baseline FFR value, patients in the Q1 and Q2 groups were 58.4 times more likely to have critical coronary stenosis than patients in the Q3 and Q4 groups. In addition, the patients in the Q1 group had a 10.23 times greater likelihood of critical stenosis compared to those in the the Q2 group; (3) the baseline FFR value was similar in all the subgroups for predicting lesion severity, and it is appropriate to use the same cut-off value for different subgroups.

FFR is the ratio of maximal blood flow to normal maximal flow in the stenosed coronary artery. It is used in the assessment of critical stenosis in the coronary arteries. It is measured as the ratio of the distal coronary pressure, obtained with a coronary pressure guidewire during CAG, to the concurrently recorded aortic pressure and is equal to 1 in a normal coronary artery. Values < 0.8 are interpreted as indicating physiologically significant stenosis and the need for revascularisation.^7^

Various agents are used to achieve maximal hyperaemia and minimal, constant coronary resistance. One of the most commonly used agents is adenosine.^8^ However, adenosine has some drawbacks. Adenosine exerts its effects on the heart through two receptors. It decreases cyclic adenosine monophosphate (cAMP) by inhibiting adenylate cyclase through its A1 receptor on the atria, ventricles, sinus node and AV node, or it increases cAMP production by activating adenylate cyclase in coronary endothelial and vascular smooth muscle cells through its A2 receptor.

The side effects of adenosine, such as transient AV block and sinus arrest, occur through stimulation of the A1 receptors, whereas effects such as coronary vasodilatation occur through the A2 receptors. Some patients may also experience dyspnoea due to activation of the chemoreceptors in the carotid sinus.^9^,^10^ Patients with afferent reflex activation may have a feeling of discomfort in the chest and chest pain that is relieved by theophylline.^11^-^13^ In some patients during FFR, adenosine has also been observed to cause ventricular arrhythmias.^14^ Furthermore, there is evidence that adenosine may be an inducer of epicardial vasoconstriction in patients with endothelial dysfunction.^15^ Consequently, adenosine has significant side effects, including angina, breathlessness, nausea and AV block, which limit its use.^16^

Given these limitations of adenosine, the initial measurement of Pd/Pa can provide an idea of the severity of the lesion without the need to use a hyperaemic agent. In a retrospective study of 520 lesions in 527 patients, three groups were formed according to baseline Pd/Pa and post-adenosine FFR measurements: a treatment group, a deferred treatment group and an indeterminate group. Patients with baseline Pd/Pa ≤ 0.86 were included in the treatment group, those with Pd/Pa of 0.87–0.99 were in the undetermined group, and those with Pd/Pa = 1 were in the deferred group. Adenosine was then administered to the indeterminate group, while those with FFR ≤ 0.80 were included in the treatment group, and those with FFR > 0.86 were in the deferral group.

In that study, which used a strategy called hybrid baseline Pd/ Pa-FFR, no significant difference in major cardiac events was observed at the five-year follow up. Therefore, adenosine could have been avoided in 14% of the patients, the positive predictive value (PPV) for FFR-guided critical lesion detection was 100% and the negative predictive value (NPV) for exclusion was 100%. It was concluded that this hybrid method may reduce the need for hyperaemia.^1^

In a single-centre retrospective study of 483 patients with 528 lesions, Mamas et al. found a strong association between resting Pd/Pa and FFR values. In their cohort, 48% of patients did not require adenosine for FFR measurement using this protocol. It was also suggested that in the case of a Pd/Pa value > 0.96, there is no need for adenosine (NPV 93%). The authors noted that the Pd/Pa ratio had a relatively high PPV (95%) and NPV (93%) in predicting a positive FFR result and stated that this finding may reduce the need for adenosine infusion.^17^

Another study found that iFR, a method of studying coronary artery pressure flow without the use of a hyperaemic agent, and baseline Pd/Pa values correlated with each other.^18^,^19^ On the other hand, in the iFR SWEDEHEART trial, the iFR-guided revascularisation strategy was non-inferior to FFR in predicting 12-month major cardiac events.^20^

In our study, the baseline FFR value, that is, the resting Pd/Pa ratio, was lower in the Q1 group, where adenosine measurements were also lower, but after adenosine infusion, it was even lower in the Q1 and Q2 groups than in the Q3 and Q4 groups. In addition, the percentage change in FFR value was significantly lower in the Q3 and Q4 groups. Therefore, according to our study, one of the indicators that the lesion is unlikely to be critical is if the baseline Pd/Pa measurement is ≥ 0.92.

The RESOLVE study was designed to evaluate the diagnostic accuracy of iFR and the Pd/Pa ratio for FFR.^2^ In that study of 1 768 patients, the primary objective was to identify specific iFR and Pd/Pa cut-off values (based on an FFR cut-off value of 0.80) and the proportion of patients above these cut-off values with 90% accuracy in predicting ischaemic and non-ischaemic FFR. For an FFR of 0.80, the best cut-off value for iFR was 0.90 (95% CI: 0.79–0.83; overall accuracy: 80.4%), and for Pd/ Pa it was 0.92 (95% CI: 0.80–0.84; overall accuracy: 81.5%), with no difference between resting measurements. iFR and Pd/Pa were 90% predictive of positive or negative FFR values in 64.9 and 48.3% of lesions, respectively. The overall precision of both non-hyperaemic indices was 80%. As a result of that study, when FFR was accepted as the reference method, iFR and Pd/Pa were found to be inadequate for guidance during the procedure, as there was a 20% discrepancy with FFR in the decisions made.^2^

In the VERIFY study, iFR was not independent of hyperaemia and correlated poorly with FFR. It was also found to be unreliable for clinical decision making in patients with coronary artery disease.^21^ In contrast to these studies, we found a significant correlation between adenosine FFR and baseline Pd/Pa measurements. During Pd/Pa measurement, the pressure in the microvascular bed is assumed to be constant at zero. Therefore, even in the presence of hyperaemia, the FFR value can be correlated with the Pd/Pa value.

Another view is that as the lesion becomes critical, vasodilatation and autoregulatory capacity in the distal part of the lesion deteriorate, so that even if a hyperaemic agent is administered, an adequate response cannot be obtained. In other words, it has been observed that the decrease in resistance during hyperaemia varies according to the severity of the stenosis.^22^ Therefore, it can be said that the baseline Pd/Pa value is proportional to the FFR value with a hyperaemic agent. The results of our study support this view.

As different patient groups have different pathophysiological processes, it is conceivable that baseline and FFR values may also be variable. For instance, it has been suggested that chronic hyperglycaemia in DM results in reduced vasodilatation and increased vascular resistance. In addition, DM has more extensive atherosclerosis with negative vessel remodelling and longer lesions.^23^ It is therefore reasonable to assume that these pathologies would affect FFR values.

In a study based on the controversial value of FFR in diabetic patients due to microvascular dysfunction, no significant difference was found between the FFR values of diabetic and non-diabetic patients with similar stenotic lesions. Furthermore, the difference in FFR before versus after adenosine infusion was similar in diabetic and non-diabetic patients.^24^

A good correlation between FFR and iFR values or resting full-cycle ratio (RFR) in both diabetic and non-diabetic patients was found in a study investigating the relationship of DM and insulin treatment with FFR and the non-hyperaemic indices iFR and RFR. A mismatch between FFR and iFR or RFR was found in approximately 20% of cases, and the frequency of mismatch was not associated with the presence of diabetes. This mismatch has been associated with microvascular dysfunction.^25^ In a trial evaluating the safety and efficacy of deferred revascularisation compared with complete revascularisation using an FFR-guided revascularisation strategy in patients with DM, major adverse cardiovascular events were more common after deferred revascularisation, especially in patients with a history of MI.^26^

A study investigating the safety of a delayed revascularisation strategy (FFR > 0.80) in patients with diabetes showed a higher rate of target lesion failure in patients with diabetes than in non-diabetic patients.^23^ On the basis of the above-mentioned studies, differences in the baseline values of Pd/Pa can be expected in patients with DM in comparison to the normal population as a result of microvascular dysfunction. However, in our study, we did not find a different predictive value for baseline Pd/Pa in patients with DM. We concluded that the previous default values can be used in patients with DM.

Of course, it is important to remember that a vulnerable plaque leading to acute coronary syndrome is not always associated with anatomically severe stenosis and that FFR measurement does not provide sufficient information about the content of the plaque. The bottom line is that even if the FFR value of patients with DM is not in the critical zone, either at baseline or with adenosine infusion, it is essential to ensure good long-term follow up, strict glycaemic control and lifestyle modification.

In a study evaluating the association between moderate CKD and FFR after stent implantation, moderate CKD was associated with insufficient improvement in FFR after stent implantation. The pre-procedure FFR values in the different eGFR groups were not significantly different.^27^ Another study, conducted to determine whether CKD (creatinine clearance < 45 ml/min) affected FFR in patients with moderate (50–70%) coronary stenosis, found that microcirculatory resistance was higher in patients with CKD, whereas a positive FFR value, defined as FFR < 0.80, was less frequent in the CKD group.^28^ Again, it is important to remember that people with CKD may have microvascular dysfunction and impaired coronary vasodilator capacity.^29^

In addition, GFR is an important indicator of kidney function, but changes in FFR measurements and different cardiovascular outcomes can also occur, for instance, in the presence of protein and albumin excretion in the urine. In a study conducted in a group of hypertensive patients, it was reported that there was a difference between normotensive and hypertensive individuals during the FFR procedure, and the FFR value was significantly lower in the hypertensive group. So, to avoid unnecessary interventions, it is important to perform measurements under conditions where blood pressure is regulated.^30^ Therefore, it can be considered that baseline Pd/ Pa values may also be affected by fluctuating blood pressure. We found no differences in baseline Pd/Pa values between different clinical conditions.

Considering this with the data in the literature, we can conclude that the adopted thresholds for the baseline Pd/Pa value can be used in different clinical groups. Clearly, more relevant data and studies are needed.

## Study limitations

There are a number of limitations in the interpretation of the study results, which should be taken into account. First, this is a single-centre, retrospective study with a relatively small number of patients. Prospective, multicentre, randomised trials with larger numbers of patients may provide more detailed results. Second, the use of additional intravascular imaging modalities in some patient groups, such as diabetic patients, may alter clinical decisions and outcomes.

Third, it is important to obtain and carefully analyse patients’ follow-up results after FFR in the medium and long term. Our study did not evaluate these data. Fourth, some of the stenoses found to be anatomically non-critical on CAG may develop into acute coronary syndrome in the future. Therefore, it is unclear whether FFR or another intravascular imaging modality should be applied to some of these non-critical stenoses. Further investigation is warranted. Beyond these limitations, our study provides important information regarding the applicability of baseline FFR cut-off values to all populations.

## Conclusion

In our study, baseline Pd/Pa correlated with FFR values during hyperaemia at certain cut-off values. No significant difference was found between baseline Pd/Pa as a reference value for lesion assessment in different clinical conditions. Therefore, baseline Pd/Pa is a parameter that does not require hyperaemia to be used in the assessment of lesion severity. In addition, the same default values for Pd/Pa can be used in different subgroups.
